# Epidemiology of vulvar cancer in Croatia

**DOI:** 10.3325/cmj.2023.64.103

**Published:** 2023-04

**Authors:** Irena Barišić, Petra Čukelj, Ivana Brkić Biloš, Mario Šekerija

**Affiliations:** 1 Croatian Institute of Public Health, Zagreb, Croatia; 2 School of Public Health Dr Andrija Štampar, School of Medicine, University of Zagreb, Zagreb, Croatia

## Abstract

**Aim:**

To assess the incidence and mortality trends of invasive vulvar cancer in Croatia between 2001 and 2019/2020.

**Methods:**

The incidence data for the period 2001-2019 were obtained from the Croatian National Cancer Registry. The number of deaths from invasive vulvar cancer by age groups between 2001 and 2020 was obtained from the Croatian Bureau of Statistics. Joinpoint regression analysis was used to assess the trends and trend changes.

**Results:**

Joinpoint regression analysis of vulvar cancer incidence rate showed a non-significant average annual percent increase (APC) of 0.8 (95% confidence interval [CI]= -0.3-2.0) during the whole period. There was also a non-significant increase in women under 60, with an average APC of 1.0 (CI= -1.6-3.7) during the whole period; similar results were obtained in women over 60 years of age (APC = 0.9; CI= -0.3-2.1). The average annual percent increase in vulvar cancer mortality rate was 0.2% (CI= -1.0-1.5), with a similar trend in women over 60 years of age (APC = 0.1; CI = -1.3-1.5). Mortality in women under 60 years of age was not assessed due to a very small number of deaths observed in the study period.

**Conclusion:**

In the studied period, the incidence of invasive vulvar cancer in Croatia was stable. Age-standardized rates (for all-ages, under 60, and over 60 years of age) increased, but the increase did not reach the level of statistical significance. The pattern in younger and older age groups was the same. The mortality rates over the last decade were stable.

Vulvar cancer is responsible for 3%-5% of all cancers of the female genital tract (1). It is a rare malignancy, with an estimated age-standardized incidence rate (ASR) of 0.85 per 100 000 women per year worldwide (2). This disease accounts for 0.5% of the total cancer cases and 0.4% of the cancer deaths in women worldwide (3).

Most malignant tumors of the vulva (80%-95%) are squamous cell carcinomas (SCC) (4). The two most frequent morphological variants of SCC are keratinizing (>60% of SCCs) and warty/basaloid (30% of SCCs) types (4). Keratinizing vulvar carcinoma may result from the progression of chronic vulvar dermatoses, such as lichen sclerosis and squamous hyperplasia. This vulvar cancer subtype, found mostly in older women, is usually not associated with the human papilloma virus (HPV). Basaloid or warty types are more common in young women, and tumor biopsies often reveal HPV DNA, especially HPV type 16 (5,6).

The incidence rates of vulvar cancer are more than 2-fold higher in high-income countries (ASR = 1.56 per 100 000 population) than in low- and middle-income countries (ASR = 0.6 per 100 000 population), while the difference in mortality rates is less pronounced (ASR = 0.35 vs ASR = 0.27). More than a third of all cases is recorded in Europe, with the highest rates in western and northern countries (2,7). The rise in the incidence of vulvar cancer in young women (under 60 years of age) is hypothesized to result from an increased exposure of this cohort to HPV infection (8-11).

According to age-standardized vulvar cancer incidence in 2020 (12), Croatia ranks tenth among EU-27 countries, while the highest rates are reported in Germany, the Netherlands, and Belgium. As for vulvar cancer mortality estimates, Croatia ranks sixth. Except in Germany, which ranks second on the vulvar cancer mortality list, the highest mortality is recorded in eastern Europe (Slovakia, Romania, Hungary, and Poland) (12).

While the incidence and mortality trends of other types of gynecological cancers (cervical, endometrial, and ovarian) in Croatia have been previously published (13), no comprehensive assessment of vulvar cancer epidemiology in Croatia has been performed. In the current study, we present the incidence and mortality trends of invasive vulvar cancer in Croatia between 2001 and 2019/2020.

## Materials and methods

The data were obtained from the Croatian National Cancer Registry (CNCR) and from the mortality database of the Croatian Institute of Public Health. The Registry, founded in 1959, covers the whole Croatian population. Data on cancer cases are collected from primary and secondary health care sources and death certificates (Croatian Bureau of Statistics), coded and analyzed at the Registry.

The analysis included all incident cases classified as C51 (malignant neoplasm of the vulva) from 2001 to 2019, according to the 10th revision of the International Classification of Disease (ICD-10). Data on patients' age, date of diagnosis, and morphological type, according to the International Classification of Diseases for Oncology, third edition (ICD-O-3), as well as information on vital status and date of death, were obtained. Patients with a morphological diagnosis of malignant melanoma (ICD-O-3 codes 8720/3, 8744/3, 8771/3) and Paget's disease of the vulva (ICD-O-3 codes 8541/3, 8542/3) were excluded from the analysis of incidence trends. We also obtained cancer registry data quality indicators: the percentage of morphologically verified cases, percentage of death-certificate-only cases, mortality/incidence ratio, and percentage of cases with no recorded stage at diagnosis. As for mortality data, only ICD-10 diagnosis was recorded, and the trends were analyzed by using the mortality data aggregated by years of death and age groups.

### Statistical analysis

Annual age-standardized (European Standard 2013) incidence rates per 100 000 women (ASIR) were calculated for all ages. Combined and truncated ASIR were derived for the age groups under 60 years and over 60 years, respectively (14). Joinpoint regression analysis was performed to assess the changes in incidence and mortality trends, for all ages and for the age groups under 60 and over 60 years (15). A p-level of 0.05 was considered significant. The analysis was conducted with Joinpoint Regression Program 4.9.1.0 (National Cancer Institute, Bethesda, MD, USA).

## Results

According to the CNCR data, between 2001 and 2019, 1451 women were diagnosed with invasive vulvar cancer. Overall, 1075 (74.1%) were diagnosed with SCC, 53 (3.7%) with either epithelial tumor other than SCC or a non-epithelial tumor, while the rest (323, 22.2%) had a tumor of unknown or unspecified histology. Regarding the patients' age at the diagnosis, 1227 (85%) were over 60 years of age and 224 (15%) were under 60 years of age. The median age at diagnosis in 2019 was 73. During the 2001-2020 period, 814 women died due to vulvar cancer, 5% of whom were under 60 years of age. Descriptive statistics on vulvar cancer incidence and mortality are presented in Table 1 and Table 2, and quality indicators for vulvar cancer data are presented in Table 3.

**Table 1:** Incidence data of vulvar cancer in Croatia, 2001-2019 period

	All ages			<60 years of age			>60 years of age		
	number	crude rate	ASR*	number	age-specific rate	truncated ASR	number	age-specific rate	truncated ASR
2001	60	2.7	1.8	11	0.7	0.6	49	8.5	8.0
2002	63	2.8	1.8	10	0.6	0.6	53	9.1	8.4
2003	75	3.4	2.2	10	0.6	0.5	65	11.1	11.1
2004	62	2.8	1.7	9	0.5	0.5	53	9.0	8.0
2005	63	2.8	1.7	8	0.5	0.4	55	9.4	8.6
2006	66	3.0	1.8	14	0.8	0.7	52	8.9	7.6
2007	77	3.4	1.9	11	0.7	0.6	66	11.2	9.2
2008	67	3.0	1.9	15	0.9	0.8	52	8.8	7.7
2009	76	3.4	2.1	10	0.6	0.5	66	11.0	10.4
2010	78	3.5	2.1	14	0.9	0.7	64	10.6	9.5
2011	74	3.3	2.0	15	0.9	0.8	59	9.7	8.3
2012	99	4.5	2.7	19	1.2	1.0	80	13.1	11.7
2013	97	4.4	2.6	18	1.1	0.9	79	12.7	11.2
2014	82	3.7	2.0	7	0.4	0.4	75	12.0	10.7
2015	95	4.4	2.1	8	0.5	0.4	87	13.7	10.9
2016	81	3.8	2.0	11	0.7	0.6	70	10.9	9.4
2017	81	3.8	2.1	16	1.1	0.8	65	10.1	8.8
2018	83	3.9	2.0	7	0.5	0.4	76	11.7	10.2
2019	72	3.4	1.9	11	0.8	0.6	61	9.3	8.5

*ASR – age standardized rate per 100 000.

**Table 2:** Mortality data of vulvar cancer in Croatia, 2001-2020 period

	All ages			<60 years of age			>60 years of age		
	number	crude rate	ASR	number	age-specific rate	truncated ASR	number	age-specific rate	truncated ASR
2001	26	1.16	0.64	1	0.06	0.05	25	4.35	3.75
2002	42	1.88	1.05	2	0.12	0.11	40	6.89	6.00
2003	36	1.61	0.93	2	0.12	0.11	34	5.82	5.24
2004	45	2.01	1.19	4	0.24	0.22	41	7.00	6.29
2005	33	1.47	0.76	1	0.06	0.05	32	5.48	4.49
2006	27	1.21	0.64	1	0.06	0.05	26	4.47	3.70
2007	44	1.97	1.02	0	0.00	0.00	44	7.49	6.39
2008	39	1.75	0.83	1	0.06	0.05	38	6.42	4.95
2009	40	1.79	0.89	3	0.18	0.15	37	6.19	4.82
2010	39	1.75	0.98	4	0.25	0.20	35	5.80	5.11
2011	34	1.53	0.78	4	0.25	0.19	30	4.93	3.93
2012	41	1.86	1.00	5	0.31	0.25	36	5.88	4.94
2013	46	2.09	1.00	1	0.06	0.06	45	7.25	5.93
2014	48	2.19	1.00	1	0.06	0.04	47	7.50	6.03
2015	48	2.21	0.92	1	0.06	0.04	47	7.43	5.50
2016	50	2.32	1.00	2	0.13	0.11	48	7.50	5.66
2017	42	1.97	0.85	3	0.20	0.16	39	6.05	4.50
2018	42	1.99	0.85	3	0.21	0.15	39	6.00	4.48
2019	51	2.43	0.97	0	0.00	0.00	51	7.77	6.08
2020	41	1.97	0.89	4	0.28	0.24	37	5.58	4.33

*ASR – age standardized rate per 100 000.

**Table 3:** Quality indicators of the Croatian National Cancer Registry data, vulvar cancer (C51)

Year	MV* (%)	DCO^†^ (%)	M/I^‡^	Unknown stage at diagnosis (%)
2001	86.7	1.7	43.3	28.3
2002	81.0	11.1	66.7	28.6
2003	85.3	6.7	46.2	26.7
2004	93.5	3.2	71.4	11.3
2005	92.1	1.6	51.6	17.5
2006	95.5	1.5	40.3	19.7
2007	71.4	9.1	57.1	39.0
2008	70.1	10.4	57.4	32.8
2009	60.5	3.9	50.0	50.0
2010	87.2	3.8	50.0	19.2
2011	78.4	2.7	45.9	24.3
2012	70.7	3.0	40.2	33.3
2013	68.0	2.1	46.5	40.2
2014	86.6	2.4	55.8	41.5
2015	88.4	5.3	49.0	42.1
2016	95.1	2.5	57.5	45.7
2017	87.7	2.5	48.3	42.0
2018	78.3	3.6	48.3	62.7
2019	79.2	2.8	64.6	65.3
Total	81.4	4.1	51.6	36.2

*percentage of morphologically verified cases.

†percentage of cases notified by death-certificate only.

‡mortality/incidence ratio (calculated including all C51 cases, irrespective of the histology).

Joinpoint regression analysis of the vulvar cancer incidence rate showed a non-significant average annual percent increase (APC) of 0.8 (95% CI= -0.3-2.0) during the whole period. There was also a non-significant change in trend in women under 60, with an average APC of 1.0 (CI= -1.6-3.7) during the whole period; similar results were obtained in women over 60 years of age (APC 0.9; CI= -0.3-2.1).

**Figure 1:**
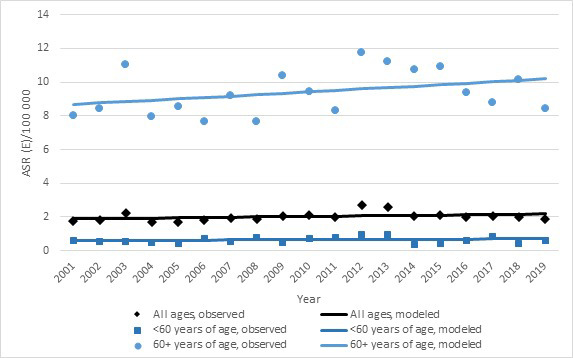
Joinpoint regression analysis results of vulvar cancer incidence rate.

**Figure 2:**
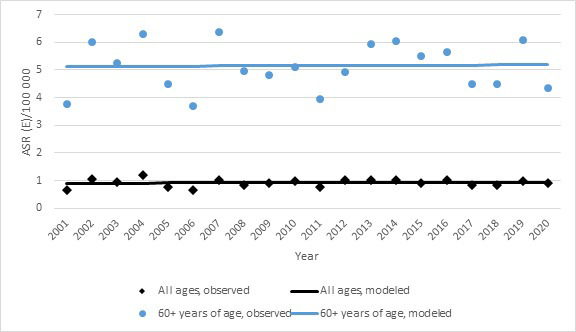
Joinpoint regression analysis results of vulvar cancer mortality rate.

Vulvar cancer mortality was also stable, both for women over 60 years of age (APC 0.1; CI= -1.3-1.5) and in the whole observed population (APC 0.2; CI= -1.0-1.5). Mortality in women under 60 years of age was not analyzed due to a very small number of cases, with zero cases of vulvar cancer deaths in some years (2007 and 2019). Joinpoint regression analysis results are presented in Figure 1 and Figure 2.

## Discussion

This is the first study to report on the epidemiological trends of invasive vulvar cancer in Croatia. Contrary to the rising incidence of this cancer in some developed European countries, our data show stable incidence trends, in both older and younger women, although an increase in age-standardized rates was observed without reaching statistical significance. Mortality trends were also stable.

Vulvar cancer incidence increases with age, with more than 50% of the patients being at least 70 years old and the median age at diagnosis being 68 years (1,16). In our study, 85% of women were above 60 years of age, and 63% were older than 70 at diagnosis. A German study (17) has reported a lower percentage (55.7%) of women diagnosed with vulvar cancer in the 70+ age group. However, it reported the highest annual increases in ASIR in women aged 30-49 years (the average APC was +9.7%). In several high-income countries, the incidence rates of SCC vulvar cancer have been increasing (7,9-11). This increase has been driven by a significant increase in incidence in women under 60 years of age, suggesting a change in HPV prevalence in younger women. This change is associated with changing sexual behaviors and increasing levels of exposure to HPV over the last several decades (2,7). Due to the aging population, we can expect that the number of women with vulvar cancer will increase in the future, as is the case with many other cancer sites. However, a part of this growth could be alleviated by HPV vaccination, especially in countries with good vaccination coverage. A recent study in Denmark showed a promising trend in the reduction of precancerous vulvar lesions in women younger than 20 and in the 20-29 age group after the implementation of the HPV vaccine in 2008 (18). However, the potential decrease in incidence rates will only be limited, as a substantial proportion of vulvar cancer cases, depending on the population in question, is not caused by an HPV infection (2,5-7).

The results of our study show a flat trend in age-standardized incidence rates of invasive vulvar cancer in the total female population, and similar results in women older and younger than 60 years. These results partially differ from the data reported by Kang et al (7). These authors assessed the incidence trends of vulvar cancer in 13 high-income countries from 1988 to 2007, with data from population-based cancer registries of nine European countries also included in the analysis. The results showed that the total invasive vulvar cancer incidence increased by 14%. The observed increase in incidence was largest in Europe (21%) and Oceania/Asia (18%). The overall rise in incidence in women under 60 was 38%; and the rise in vulvar cancer incidence in all age groups in Denmark, Germany, UK, and the Netherlands was driven mainly by the increase in this age group. Our data demonstrated no such increase in this age group. However, the negative medical impact of previous exposure to HPV in women younger than 60 in Croatia may be yet to come. Due to vulvar cancer being a rare entity and the relatively small population of Croatia, it is difficult to recognize any possible significant trends in this population.

Contrary to the increase in vulvar cancer incidence in younger women, Kang et al (7) found no significant change among women older than 60 years of age. The only European countries with an increase in vulvar cancer incidence in this age group were Germany and the Netherlands, where an especially high vulvar cancer incidence was reported (17). Our results demonstrated a fluctuation of incidence rates in the 60+ age group, with the increase in incidence not reaching statistical significance.

One of the few well-established risk factors for vulvar cancer (especially in younger women) is HPV infection (7,8). HPV prevalence is highest in northern Europe, somewhat lower in the central part of the continent, and lowest in southern European countries (19). This north-south gradient could partly explain the significant increase in vulvar cancer observed in western Europe. No difference in vulvar cancer risk was found for the majority of sexual behavior determinants (19).

Despite the introduction of less radical surgical techniques and more individualized treatment options with the aim to reduce morbidity, the current study showed a stable trend of mortality rates. These results agree with those reported for the Netherlands (11), but are less ominous than the increase in the age-standardized rates in Germany (from 0.7 per 100 000 women in the period 1999-2002 to 0.9 per 100 000 women in the period 2009-2012) (16). Nevertheless, an increase in overall incidence but a decrease in mortality of women aged over 60 in the first decade of the 21st century in England (20) has announced some promising trends.

This is the first report on the epidemiological trends of vulvar cancer in Croatia and it is necessary to acknowledge some important limitations of the study. First, the data in the Croatian National Cancer Registry are not yet of sufficient quality regarding histological types and stage at diagnosis of vulvar cancer. For this reason, we did not present the data on the stage distribution of the disease, which would be beneficial to further describe the progress in vulvar cancer awareness and diagnosis. Although the basic descriptive data on histology are presented, a further analysis was not performed due to many cases with unknown or unspecified histology of the tumor.

In conclusion, more awareness in the field of vulvar cancer is needed, from both medical professionals and women in order to recognize the symptoms and the importance of regular check-ups. This could lead to earlier diagnosis and a decrease in mortality.
